# The effects of error-augmentation versus error-reduction paradigms in robotic therapy to enhance upper extremity performance and recovery post-stroke: a systematic review

**DOI:** 10.1186/s12984-018-0408-5

**Published:** 2018-07-04

**Authors:** Le Yu Liu, Youlin Li, Anouk Lamontagne

**Affiliations:** 1 School of Physical and Occupational Therapy, McGill University, 3654 Promenade Sir-William-Osler, Montréal, Québec H3G 1Y5 Canada; 2 Feil and Oberfeld Research Centre, Jewish Rehabilitation Hospital site of CRIR (CISSS Laval), Laval, Canada

**Keywords:** Cerebrovascular accident, Upper extremity, Robotics, Error-augmentation, Error-reduction, Haptic guidance, Evidence, PEDro

## Abstract

Despite upper extremity function playing a crucial role in maintaining one’s independence in activities of daily living, upper extremity impairments remain one of the most prevalent post-stroke deficits. To enhance the upper extremity motor recovery and performance among stroke survivors, two training paradigms in the fields of robotics therapy involving modifying haptic feedback were proposed: the error-augmentation (EA) and error-reduction (ER) paradigms. There is a lack of consensus, however, as to which of the two paradigms yields superior training effects. This systematic review aimed to determine (i) whether EA is more effective than conventional repetitive practice; (ii) whether ER is more effective than conventional repetitive practice and; (iii) whether EA is more effective than ER in improving post-stroke upper extremity motor recovery and performance. The study search and selection process as well as the ratings of methodological quality of the articles were conducted by two authors separately, and the results were then compared and discussed among the two reviewers. Findings were analyzed and synthesized using the level of evidence. By August 1st 2017, 269 articles were found after searching 6 databases, and 13 were selected based on criteria such as sample size, type of participants recruited, type of interventions used, etc. Results suggest, with a moderate level of evidence, that EA is overall more effective than conventional repetitive practice (motor recovery and performance) and ER (motor performance only), while ER appears to be no more effective than conventional repetitive practice. However, intervention effects as measured using clinical outcomes were under most instance not ‘clinically meaningful’ and effect sizes were modest. While stronger evidence is required to further support the efficacy of error modification therapies, the influence of factors related to the delivery of the intervention (such as intensity, duration) and personal factors (such as stroke severity and time of stroke onset) deserves further investigations as well.

## Background

Stroke, also referred to as cerebrovascular accident (CVA), is one of the leading causes of disablement among adults [1, 2]. It is estimated that stroke costs the Canadian, United States and United Kingdom economy around $3.6 billion [3], $34 billion [4] and £9 billion [5] a year respectively in medical services, personal care and lost productivity. The disabilities resulting from stroke can affect all aspects of life including gross and fine motor ability, walking, activities of daily living (ADLs), speech and cognition [6]. Motor impairments are some of the most prevalent issues post stroke and restoring upper extremity function is one of the top priorities of people with stroke [7]. Compared to the lower extremity impairments, the upper extremity impairments are more likely to result in activities limitations (see International Classification of Functioning, Disability and Health (ICF) in Appendix 1) because tasks that involve the arm and hand often require a high level of fine motor control [8]. In fact, severe upper extremity impairments post-stroke often hinder the ability to take care for oneself and perform ADLs [9]. Although restoration of upper extremity motor functions is crucial for stroke patients to regain their independence, studies have shown that only 35 to 70% of people with stroke recover to the level of arm ability that is considered functional [10–12] while more than 50% have persistent upper extremity impairments [13].

Studies in both human and animal models demonstrate the importance of motor learning in the process of motor recovery following an acquired brain lesion as both learning and recovery processes can induce cortical changes and reorganization [14]. Motor learning, which is “a set of processes associated with practice or experience that leads to relatively permanent changes in the ability to produce skilled action” [15], relies on an experience-dependent neural plasticity that is modulated by various factors such as task specificity, repetition, intensity, timing, salience, etc. [16]. Amongst different factors influencing the acquisition of motor skills, feedback is believed to be one of the key factors [15]. Feedback is the information that an individual receives as a result of his or her performance [17]. It can be either intrinsic or extrinsic, where intrinsic feedback is that experienced by the performer (e.g. sensory, visual feedback, etc.) and extrinsic (augmented) feedback is that provided by an external source, such as a therapist providing verbal or physical guidance [18, 19]. Extrinsic feedback can inform the performer about a success or failure on a task (knowledge of results) or about the quality of movement or task performance (knowledge of performance) [15].

Robotics is one of the advanced technologies that is increasingly used in post-stroke upper extremity rehabilitation [20]. Compared to conventional approaches, it offers the advantages of high convenience when providing task-oriented practice, as well as high accuracy in measuring outcomes of motor performance (e.g. trajectory straightness, movement speed, range of joint movement [21]). The latter outcomes can in turn be used to provide knowledge of performance as a source of feedback [22]. Two main paradigms of training on the use of feedback, arising from the literature on robotics, were proposed and tested as means to facilitate motor learning and improve motor performance: the error reduction (ER) paradigm and error augmentation (EA) paradigm. The ER paradigm, also known as haptic guidance, is to reduce the performance errors of a subject during a motor task [23], namely via the assistance provided by a robot so that the performer can stay within the optimal movement trajectory determined by the non-paretic arm or by the therapist [24]. This approach is based on the hypothesis that by demonstrating the correct movement trajectory to a person, he/she will be able to learn it by imitation [25]. The discovery of “mirror neurons” that were first identified using microelectrode recordings of single neurons in area F5 of monkey premotor cortex [26] prompted the researchers to believe that a similar mirror neuron system exists in humans, and that this mirror neuron system could play an important role in learning through imitation [27]. Furthermore, the theory of reinforcement-based learning suggests that positive/successful feedback is essential for motor learning to occur [28]. The ER paradigm also assumes that there is a unique optimal movement trajectory and any deviation from it is considered to be an error. According to the principle of abundance and the theory of use-dependent learning, however, having variance in how a motor action is performed does not necessarily impede the overall motor performance [29, 30].

A whole body of literature also suggests that motor learning can be an error driven process, a postulate that can be explained and supported by motor control theories such as the internal model theory [31] and the equilibrium point hypothesis [32]. In the internal model theory, it is hypothesized that subjects form an ‘internal model’ based on their anticipation of the effects of the environment on their motor actions, therefore the internal model acts as a feed-forward component of the motor control [31]. The detection of errors that occur during the motor performance play the role of a feedback component, as errors prompt the existing internal model to adapt in order to reduce errors [33–36]. In the equilibrium point hypothesis, the errors occur in the subsequent movements following a change in the environment, but the motor system is able to correct these errors by adjusting the control variables based on information about the current motor system, joint positioning of the limbs, etc., thus resetting the activation thresholds (λ) of muscle and forming a new equilibrium point [32, 37]. Given the role of errors in motor learning, it was hypothesized that artificially increasing the performance error would cause learning to occur more quickly [25], an idea that is the foundation of the EA paradigm. In robotics, one of the commonly used technique to artificially increase performance error is to create a force-field that disturbs the limb motion during the movement [38].

While the theories and ideas that support ER vs. EA paradigms are distinct, both are currently being used, primarily in the form of haptic feedback, as part of clinical intervention studies for populations with deficits in motor recovery. Until this day, there is no consensus as to which of the two paradigms provides superior treatment effects in upper extremity motor recovery and performance among stroke survivors. Furthermore, while systematic reviews on the use of error modification in upper extremity rehabilitation after stroke were published in the recent years [39, 40], these exclusively focused on the EA paradigm and did not allow for a comparison between the two approaches. In this study, we conducted a systematic review on the use of EA and ER paradigms in the form of haptic feedback to enhance upper extremity motor recovery and performance in stroke survivors. The main research questions that were addressed are listed in PICO format (Population, Intervention, Comparison, and Outcome) and read as follows:

1. Among stroke survivors (P), to which extent do interventions involving EA paradigm (I_1_) or ER paradigm (I_2_) compared to interventions without error modification (C) enhance the upper extremity motor recovery and performance respectively (O).
2. Among stroke survivors (P), to which extent does the EA paradigm (I) compared to ER paradigm (C) enhance the upper extremity motor recovery and performance (O).

For the purpose of clarification, the comparison component of the first research question, “training without error modification,” refers to standard repetitive practice that does not involve any external force (reducing or amplifying errors) that provides feedback on the performance. The outcomes of both research questions, “upper extremity motor recovery and performance,” can include clinical measures of both upper extremity impairment and disability and kinematic measures of motor performance (for more details, refer to the section of inclusion and exclusion criteria).

## Methods

### Search strategy

The following databases which are available through McGill University library were systematically searched using their online search engines: Ovid MEDLINE, CINAHL, EMBASE, AMED, PsychoInfo, and PEDro. There was not a start date limit on the search criteria of the database, and the end date was August 1st 2017. The overall search strategy which was determined by the two reviewers (L.Y.L. and Y.L.) involved multiple search entries with keywords listed in the following, and the corresponding Medical Subject Headings (MeSH) terms were selected and ‘exploded’ (* for truncation):

- Search 1: error amplifica*, error augment*, error enhance*, error enhancing, negative viscosity, haptic guidance, haptic*, active assist* (all keywords were combined with OR operator).
- Search 2: stroke/ or stroke rehabilitation (MeSH), post-stroke (all keywords were combined with OR operator).
- Search 3: upper extremity/or arm (MeSH), upper-extremity, upper arm, motor learn*, reaching (all keywords were combined with OR operator).
- Final search: all three previous searches were combined with AND operator.

Following the electronic database search, a manual search of all relevant studies was performed to ensure the completeness of the search.

### Study selection process

All search results found in the databases were saved into EndNote X7 reference manager (1988–2013 Thomson Reuters), and the duplicates were removed by the software. Each of the two reviewers carried out the study selection process separately. In addition, the study selection process involved the following steps: (1) Screen the remaining articles by their titles and abstracts; (2) Remove studies that do not meet the inclusion criteria or meet the exclusion criteria; (3) Review the full text of the remaining articles and; (4) Remove studies that do not meet the inclusion criteria or meet the exclusion criteria. Following step 4, the two reviewers compared their results. They discussed about the discrepancy between the results and decided together which articles were to be selected and the process of data extraction began.

### Inclusion and exclusion criteria

The following were the inclusion criteria:

1. The population of the study is people with stroke who have upper extremity hemiparesis. The severity and onset of stroke may vary.
2. The design of the studies can be randomized controlled trial, crossover trials, quasi-experimental trials and pilot studies. The studies have to be intervention-oriented and not observation-oriented or review-oriented.
3. The upper-extremity tasks involved in the experimental procedure can be reaching, moving arm in circular trajectory, timing-oriented, grasping, or other functional movements.
4. The interventions of the studies have to involve either EA, ER or both paradigms. If the interventions only contain feedback and not any error modification, they are not included.
5. The interventions have to be mainly based on haptic feedback, but other feedback such as visual and auditory can be used as supplement. The reason to focus on haptic feedback is because based on previous review papers, most studies on EA and ER paradigms were in the field of robotics and haptic feedback was mainly involved. Therefore, to facilitate the comparison process only studies involving haptic feedback are included.
6. The studies can either compare EA to ER or compare either EA or ER to standard repetitive practice training that does not involve error modification.
7. The outcomes of the studies can be either kinematics or clinical outcomes. The kinematic outcomes have to measure the quality of movement such as trajectory straightness, smoothness, timing error, etc. The clinical outcomes can measure either the impairment level (i.e: range of motion, spasticity, level of motor recovery) or the motor disability level. The assessment tools have to be validity-proven such as Fugl-Meyer Assessment [41], Chedoke-McMaster Stroke Assessment [42], etc.

The following were the exclusion criteria:

1. The language of publication is not English.
2. The age of population studied is under 21 years old. Stroke in pediatric population may differ in aetiology, presentation and response to intervention and including this age range could introduce several confounding variables in this study.
3. The number of participants is less than 5, in order to control the statistical certainty of the results. Therefore, case studies are excluded.
4. The articles that are listed as conference abstracts are excluded.
5. The main outcomes are not related to motor performance (as defined in the introduction) or recovery of upper extremity.

### Methodological quality assessment

The Physiotherapy Evidence Database (PEDro) scale [43] was chosen for the quality assessment of all articles selected, as studies have shown that the validity and reliability of PEDro scale are well established [44–46]. The scale consists of 11 items: eligibility criteria specified, randomized allocation, concealed allocation, baseline similarity, blinded subjects, blinded therapists, blinded assessors, adequate follow-up, intention to treat analysis (an analysis was performed as if the subjects received the treatment as allocated even if they received a different treatment), comparison between groups, point estimates and variability [45]. One point is awarded when a criterion is clearly satisfied, except the first criterion ‘eligibility criteria specified’ which is not considered for the calculation of score, therefore the total score is out of 10. PEDro scores are interpreted as follows: 6–10 indicates high methodological quality, 4–5 corresponds to fair quality, and less than 4 indicates poor quality [47]. The two reviewers (L.Y.L and Y.L) rated each of the selected studies separately, and the agreement among the two was calculated using Cohen’s kappa for each of the eleven items of PEDro scale. Then they compared and discussed their scores to decide the final score for each of the articles.

### Risk of bias assessment

The risk of bias was evaluated using the Cochrane Collaboration’s risk of bias tool [48] by the reviewer L.Y.L. This tool was developed in 2005 by the Cochrane Collaboration’s Methods Group as the new strategy for addressing the quality of randomized trials [49]. The Cochrane Collaboration’s risk of bias tool involves the assessment of the risk of bias arising from each of six domains: random sequence generation, allocation concealment, blinding of participants and personnel, blinding of outcome assessment, incomplete outcome data, selective reporting and other biases [48, 49].

### Data extraction

The studies selected were divided into three categories based on their interventions and comparisons: (1) EA compared to training without error modification, (2) ER compared to training without error modification and (3) EA compared to ER. For each category of studies, a description table was used. The following data were abstracted from the selected studies:

- Study design, and in case of a clinical trial, indicating if the trial is registered in ClinicalTrials.gov (run by the United States National Library of Medicine)
- Number of participants in the experimental and control groups
- Demographic and clinical information of the participants
- Equipment used
- Experimental protocol including the parameters of training
- Main outcomes measures and assessment tools used
- Results of the study including the significant levels and interpretations
- Effect sizes of the results
- Methodological quality scores of the study calculated using the PEDro scale.

### Data analysis and synthesis

Outcomes were considered as significant if: (1) the reported *p*-value was less than 0.05 or (2) the 95% confidence interval did not contain 0. To calculate the effect size, the Cohen’s *d* formula: *d* = Mean_group1_-Mean_group2_/standard deviation_pooled_ was used. If *d* is between 0.2 and 0.5, the effect size was considered small; between 0.5 and 0.8, it was medium and above 0.8, it was large [50]. If the numerical values of the results were not reported in a particular study, a textual explanation would be stated in the results column or effect size column of the tables. In order to synthesize the results, ratings of level of evidence from Evidence Based Medicine were used (Appendix 3) [51].

## Results

### Study selection

**Fig. 1:**
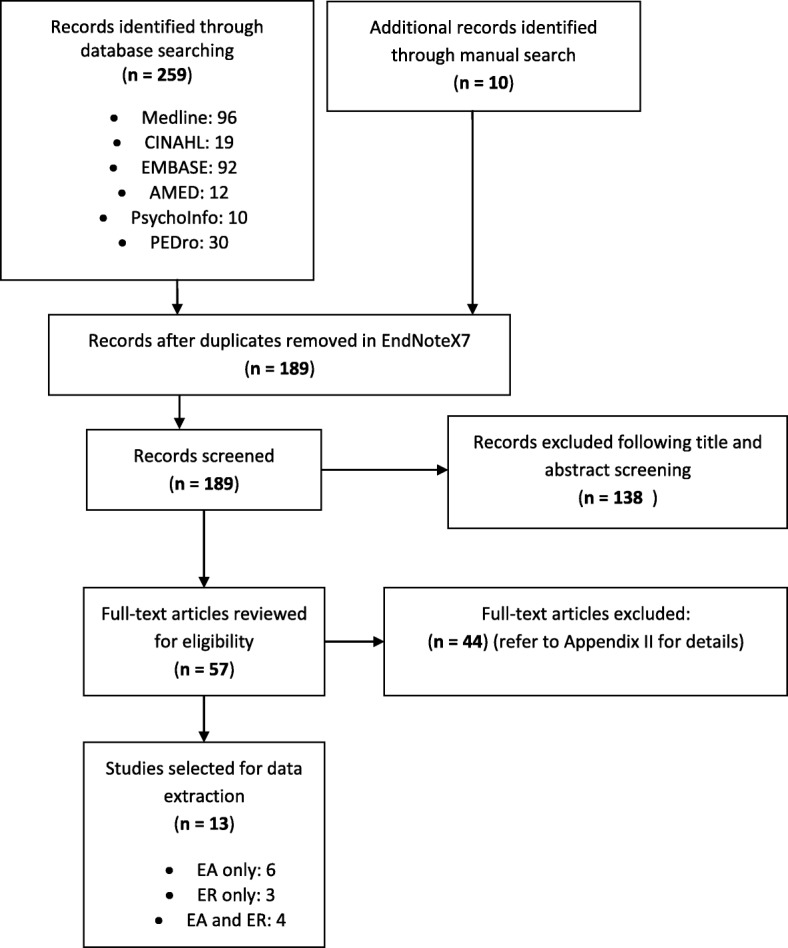
The selection process of studies using PRISMA 2009 flowchart

Figure 1 illustrates the selection process of the studies included in this paper using the PRISMA 2009 flowchart. The overall search results consisted of 259 articles from the databases, and 10 from the manual search. Among the 269 studies, 80 duplicates were removed using EndNote X7, and 138 were excluded based on title and abstract screening. Furthermore, following full text reviews, 44 studies were excluded (see Appendix 2) such that 13 remaining articles were retained for the data extraction and synthesis. Among these 13 articles, 6 compared the effects of EA to training without error modification, 3 compared the effects of ER to training without error modification, and 4 compared EA to ER.

### Study designs

Table 1 (EA only), Table 2 (ER only) and Table 3 (EA and ER) described all 13 studies as well as their results. Among the 13 selected studies, there are six randomized controlled trials (RCT) [52–57], five crossover studies [52, 54, 58–60], one quasi-experimental study [24], two randomized comparative study [61, 62] and one pilot study [63]. Among all thirteen studies, only four could be found in clinical trials registry [52, 56, 57, 63].

**Table 1:** Summary of studies that compared EA to training without error modification

Article	Study design	Number of participants: experimental group (E) and control group (C)	Participants characteristics	Equipment	Experimental Protocol	Outcomes and assessment tools	Main results and interpretation (means ± standard deviation)	Effect size (Cohen’s *d*)	Quality of study (PEDro score)
Abdollahi et al. [52]	Crossover randomized controlled trial. Trial registered NCT01574495	27 in total, E = 13, C = 14 (before crossover)	Ages: 36–88 years (mean = 57.92 ± 9.96), 12 males and 15 females, all participants suffered a single cortical or subcortical stroke at least 6 months prior to the study (mean = 82.34 ± 72.04 months, 9 hemorrhagic, 18 ischemic) FM^b^ scores: 15–50 (mean/SD unknown)	Virtual Reality Robotic and Optical Operations Machine (VRROOM). Phantom Premium 3.0 robot.	Experimental task: various reaching movement Control group: received only treatment of repetitive practice EA group: same as the control group in addition of combined visual and haptic error augmentation Training parameters: 60 min per session, three sessions per week, two weeks of training per phase (two). After first phase, all subjects switched to the other group.	Clinical ROM^c^, AMFM^a^, WMFT FAS^d^, WMFT time measure, Box and Blocks test	ROM: no significant effects AMFM: in the first phase, EA showed more improvement than control (2.08 ± 2.25 vs 0.69 ± 2.90), and this difference was significant [F(1,24) = 4.261, *p* < 0.05] In the second phase, EA was still better than control (1.15 ± 2.21 vs 0.54 ± 2.30), but not significantly (numerical values not provided) WMFT FAS: in the first phase, EA was better than control (0.11 ± 0.24 vs 0.01 ± 0.16), but level of significance unknown. In the second phase, control was better than EA (0.14 ± 0.22 vs − 0.02 ± 0.25), but level of significance unknown. WMFT time: in the first phase, EA was better than control (1.48 ± 8.86 vs − 0.53 ± 5.19), but level of significance unknown. In the second phase, EA was better than control (1.19. ± 5.68 vs 0.17 ± 8.02), but level of significance unknown. Box and Blocks test: no significant effects	AMFM: first phase 0.53 (medium effect). second phase 0.27 (small effect). WMFT FAS: first phase 0.51 (medium effect). second phase 0.52 (medium effect) WMFT time: First phase 0.28 (small effect) Second phase 0.14 (very small effect)	7/10, high quality
Givon-Mayo et al. [63]	Pilot study. Trial registered NCT02017093	7 in total, E = 4 and C = 3	Ages: 45–78 (mean = 59.14 ± 9.77), 8 males and 1 female. All participants sustained a stroke (1 hemorrhagic, 8 ischemic) 2 to 3 weeks prior to the study. FM scores: EA group mean = 53.25 ± 3.77 Control group mean = 54.33 ± 3.84	DeXtreme prototype robot- 2 degrees of freedom. Free end Robot.	Experimental tasks: reaching EA group: received error inducing forces from the robot Control group: also attached to the robot, but did not receive forces Training parameters: 20 min per session, three sessions per week for five weeks	Kinematic: movement velocity deviation error (cm/sec). Clinical: FM, *MAS*^e^	*MAS*: the EA group showed more improvement than the control group (3.2 ± 2.6 vs 1.7 ± 3.2), but the level of significant unknown FM: no significant changes Velocity deviation error: the EA group showed significant (*p* < 0.05) more improvement than the control group (− 16.8 ± 3.8 vs − 4.7 ± 3.8)	*MAS*: 0.51 (medium effect) Velocity deviation error: 3.2 (very large effect)	3/10, poor quality
Huang and Patton [59]	Crossover design. Trial not registered	30 in total, Participants were randomly assigned to either one control group or two experimental groups, but the numbers are unknown.	Mean age = 52.0 ± 8.2, all participants suffered from a chronic stroke (mean = 102.0 ± 84.0 months). Clinical assessment results prior to the study were not available	A planar force feedback device. The subject’s arm was supported by a low-friction, low-impedance mechanism	Experimental tasks: circular movement task Control group practiced on the training device in null-field conditions in all sessions. The two experimental groups: trained in a null field condition in the first session, then received either EA force alone or EA force combined with positive limb inertia in the next session. They switched to the other condition in third session Training parameters: Two hours per session, three sessions in total	Kinematic: radial deviation (distance between the handle and template circular track) (mm)	When evaluated in the next session, the control showed no significant improvement (0.7 mm ± 2.3, 95% confidence interval: − 0.4 to 1.8). The EA group showed the largest significant improvement (1.4 ± 2.7, CI: 0.2 to 3.0) while the combined EA with inertia group showed non-significant improvement (1.1 ± 2.7, CI: 0 to 2.2)	EA compared to control: 0.28 (small effect). EA compared to combined EA with inertia: 0.11 (very small effect)	3/10, poor quality
Majeed et al. [62]	Randomized comparative experiment. Trial not registered.	28 in total, participants were randomized into experimental and control groups based on blocks of FM scores	Ages: 26–78 (mean = 55.38, SD unknown), 17 males and 11 females, all participants suffered from a cortical chronic stroke (mean/SD unknown), Upper extremity FM score: 25–49 (block randomized into both groups, mean/SD unknown)	Three-dimentional haptic/ graphic system called the Virtual Reality Robotic and Optical Operations Machine (VRROOM)	Experimental tasks: reaching Control group: received only treatment of repetitive practice EA group: same as the control group in addition of combined visual and haptic error augmentation Training parameters: 45 min per session, three sessions per week, two weeks of training	Clinical: AMFM	At the end of 2 weeks of training, no significant difference was found between EA and control groups in improvement of AMFM (numerical data not provided). At one-week follow-up, EA group showed more retention than control group (2.60 ± 3.50 vs − 0.1 ± 6.98), but the level of significant known	From the end of training to one-week follow-up: 0.52 (medium effect)	6/10, high quality
Patton et al. [53]	Randomized controlled trial. Trial not registered	15 in total. E = 12, C = 9 (6 subjects returned for a second visit, so they served as their own control)	Ages: 30–76 (E: mean = 50.66 ± 13.08; C: mean = 50.77 ± 12.16), 9 males and 6 females, all participants suffered from a chronic stroke (E: mean = 77.25 ± 40.85 months; C: mean = 99.89 ± 44.13) prior to the experiment. FM score (E: mean = 34.36 ± 12.23; C: mean = 35.0 ± 11.79	A two degrees-of-freedom robot	Experimental tasks: reaching EA group: received EA forces from the robotic while doing repetitive practice. Control group: Same training but without EA forces. Training parameters: One single session of three hours and consisted of 744 movements	Kinematic: size of movement error (change in %) Clinical: AMFM	AMFM: the EA group had a greater improvement than control group (1.6 ± 2.6, *p* = 0.06 vs 0.4 ± 1.1, *p* > 0.27), but the results were not significant Movement error size: the EA group had a greater improvement than control group (− 45.2 ± 80.6 vs − 11.1 ± 48), but the levels of significance unknown	AMFM: 0.65 (medium effect) Movement error size: 0.53 (medium effect)	5/10, fair quality
Rozario et al. [60]	Crossover design. Trial not registered	10 in total Stroke group (before cross-over) EA = 3 Control = 2 Healthy group 5	Stroke: ages: 36–69 (mean = 55.0 ± 12.1), 4 males and 1 female, suffered a single cortical stroke for more than 6 months (mean/SD unknown), AMFM: EA group mean = 35.33 ± 8.14 Control group mean = 43.50 ± 2.12 Healthy: ages: 19–27 (mean/SD unknown)	A 6-degree of freedom PHANTOM Premium 3.0 robot	Experimental tasks: various reaching movement. Control group: received only treatment of repetitive practice EA group: same as the control group in addition of combined visual and haptic error augmentation Healthy group: did not receive any treatment, only data collected Training parameters: 40 min per session, three sessions per week, and two weeks per phase (two). After first phase, all subjects switched groups.	Kinematic: ROM errors (m) Clinical: AMFM, WMFT FAS, WMFT time, Box and Blocks test	Clinical test: no noticeable changes in any of the clinical tests (numerical data not provided). ROM errors: In the first phase, EA showed more improvement than control group (0.08 ± 0.08 vs 0.04 ± 0.04), level of significant unknown. In the second phase, EA showed no improvement but control group showed deterioration (0 ± 0 vs − 0.02 ± 0.03). More errors seen in stroke subjects than healthy subjects.	ROM errors: first phase 0.75 (medium effect). Second phase 1.05 (large effect)	4/10, fair quality

^a^EA/ER: error augmentation/error reduction

^b^FM/AMFM: Fugl-Meyer assessment/Arm Motor Fugl-Meyer

^c^ROM: Range of motion

^d^WMFT FAS: Wolf motion function test-functional ability scale

^e^MAS: Motor Assessment Scale

**Table 2:** Summary of studies that compared ER to training without error modification

Article	Study design	Number of participants: experimental group (E) and control group (C)	Participants characteristics	Equipment	Experimental Protocol	Outcomes and assessment tools	Main results and interpretation (means ± standard deviation)	Effect size (Cohen’s *d*)	Quality of study (PEDro score)
Kahn et al. [55]	Randomized controlled trial. Trial not registered	19 participants with stroke in total E = 10 C = 9	E: age (mean) = 55.6 ± 12.2, four males and six females, months post-stroke (mean) = 75.8 ± 45.5, CM^c^ (mean) = 3.5 ± 0.9 C: age (mean) = 55.9 ± 12.3, seven males and two females, months after stroke (mean) = 103.1 ± 48.2, CM (mean) = 3.2 ± 1.0	ARM Guide (the Assisted Rehabilita-tion and Measure-ment Guide), a robotic device that drives user’s hand along a linear rail	Experimental task: reaching ER^a^ group: the subjects received robotic active assistance Control group: they were not attached to the device Training parameters: A single session consisted of 50 trials	Kinematic: Supported fraction of range (FR_s_), supported fraction of speed (FS_s_), unsupported fraction of speed (FR_u_). Clinical: CM	FR_s_: ER group showed more improvement than control group (0.14 ± 0.11 vs 0.12 ± 0.09), but not significantly (*p* = 0.844) FS_s_: ER group showed more improvement than control group (0.22 ± 0.09 vs 0.21 ± 0.13), but not significantly (*p* = 0.898) FR_u_: control group showed more improvement than ER group (0.02 ± 0.07 vs 0.01 ± 0.09), but not significantly (*p* = 0.687) CM: control group showed more improvement than ER group (0.3 ± 0.5 vs 0.2 ± 0.4), but not significantly (*p* = 0.414)	FR_s_: 0.2 (small effect) FS_s_: 0.09 (very small effect) FR_u_: 0.13 (very small effect) CM: 0.22 (small effect)	4/10, fair quality
Takahashi et al. [56]	Randomized controlled trial Trial registered NCT01244243	13 participants with stroke in total Group A-A: 7 Group ANA-A: 6	A-A group: age (mean) = 58.6 ± 16, months since stroke (mean) = 14.4 ± 13.2 AMFM^b^ = 40.4 ± 10.5 ANA-A group: age (mean) = 67.3 ± 15, months since stroke (mean) = 57.8 ± 87.6, AMFM = 49.5 ± 8.6	HWARD (The Hand Wrist Assistive Rehabilita-tion Device) is 3 degrees of freedom that assists the hand in grasp and in release movement	Experimental task: Grasp and release exercises A-A (Active Assist) group: the subjects received robotic assistance throughout the training sessions. ANA-A (Active Non-Assist to Active Assist) group: the subjects were attached to the robotic device but did not receive assistance for the first half of the treatment phase, then received robotic assistance in the second half of the phase. Training parameters: 15 sessions over three weeks. Each session lasted around one hour and half.	Clinical: ARAT^d^, AMFM, Box and Blocks test Other: EMG^f^, fMRI^g^	ARAT: by the end of session, the A-A group showed more improvement than ANA-A group (5.3 ± 2.1 vs 2.8 ± 1.8), the difference was significant [F(2,10) = 5.2, *p* < 0.03]. AMFM:: by the end of session, the A-A group showed more improvement than ANA-A group (9.1 ± 2.1 vs 5.8 ± 1.6), the difference was significant [F(2,10) = 4.8, *p* < 0.04] Box and Blocks: no significant difference found between the two groups EMG: no significant change within subjects in the recruitment pattern of muscle fMRI: the percent signal change in the left primary sensorimotor cortex remained stable (*p* > 0.3)	ARAT: 1.28 (very large effect) AMFM:1.78 (very large effect)	5/10, fair quality
Timmer-mans et al. [57]	Randomized controlled trial. Trial registered on *ISRCTN registry* ISRCTN82787126	22 participants with stroke in total E = 11 C = 11	E: age (mean) = 61.8 ± 6.8, eight males and three females, months post-stroke (mean) = 44.4 ± 36, AMFM (median) = 53 C: age (mean) = 56.8 ± 6.4, eight males and three females, years after stroke (mean) = 33.6 ± 34.8, AMFM (median) = 50	The robotic system Haptic Master, with six degrees of freedom	Experimental task: functional tasks (‘drinking from a cup,’ ‘eating with a knife and fork,’ ‘taking money from a purse’ or ‘using a tray.’ ER group: subjects received trajectory guidance from the robot. Control group: the subjects were not supported by the robot. Training parameters: Two sessions of 30 min (break of 0.5 to 1 h) per day, four days per week for eight weeks.	Clinical AMFM, ARAT, MAL AU and QU^e^	AMFM: by the end of 8 weeks training, the control group showed more improvement than ER group (3.5 ± 5.9 vs 1.6 ± 17.9), but the result was not significant (*p* = 0.51). ARAT: the control group showed more improvement than ER group (16.1 ± 26.5 vs 9.0 ± 11.0), but the result was not significant (*p* = 0.79). MAL AU: the control group showed more improvement than ER group (33.1 ± 64.0 vs 9.0 ± 37.5), but the result was not significant (*p* = 0.33). MAL QU: the control group showed more improvement than ER group (46.5 ± 40.9 vs 41.3 ± 33.1), but the result was not significant (*p* = 0.4).	AMFM: 0.16 (very small effect) ARAT: 0.38 (small effect) MAL AU: 0.47 (small effect) MAL QU: 0.14 (very small effect)	8/10, high quality

^a^EA/ER: error augmentation/error reduction

^b^FM/AMFM: Fugl-Meyer assessment/Arm Motor Fugl-Meyer

^c^CM: Chedoke-McMaster scale score

^d^ARAT: Action Research Arm Test

^e^MAL: Motor Activity Log, AU: Amount of use, QU: Quality of use

^f^EMG: Electromyography

^g^fMRI: Functional Magnetic Resonance Imaging

**Table 3:** Summary of studies that compared EA to ER

Article	Study design	Number of participants: experimental group (E) and control group (C)	Participants characteristics	Equipment	Experimental Protocol	Outcomes and assessment tools	Main results and interpretation (means ± standard deviation)	Effect size (Cohen’s *d*)	Quality of study (PEDro score)
Bouchard et al. [61]	Randomized comparative experiment. Trial not registered.	34 in total EA group: 17 haptic guidance (ER) group: 17	ER group: age (mean) = 67 ± 7, months since stroke (mean) = 63 ± 54 AMFM^a^ = 63 ± 8 EA group: age (mean) = 67 ± 6, months since stroke (mean) = 78 ± 64 AMFM = 60 ± 10	TEO, a robotic device with 10-degree flexion/ extension of the left/ right wrist, actuated by Dynamixel MX-106 servomotor	Experimental task: flex paretic wrist at the right time. ER group: the robotic device adjusted its activation time to reduce the timing errors (k value decreased by 90%). EA group: the opposite, the timing errors were increased (k value increased by 90%) Training parameters: Four phases of baseline assess-ment (140 trials) before the intervention. 75 trials during the inter-vention, and 40 trials at the reten-tion phase.	Kinematic: Absolute timing errors (ms)	A significant decrease of 1.1 ± 5.1 ms in absolute timing errors in the ER group (*p* = 0.032), and a non-significant increase of 0.4 ± 6.0 in the EA group (*p* = 0.45). A between group comparison revealed no significant difference between the two groups (95% confidence interval: − 1.2 to 4.3)	0.27 (small effect size)	8/10, high quality
Cesqui et al. [58]	Crossover design. Trial not registered	15 in total EA group: 6 ER group: 9 (before cross-over)	Ages: 20–71 years (mean = 42 ± 17) 8 males and 7 females, all participants suffered from stroke (stages unknown) CM^b^: First EA group: mean = 5 ± 0.89 First ER group: mean = 4 ± 0.86	InMotion2	Experimental tasks: reaching targets in a plane. EA group: received divergent field (negative elastic force) ER group: received active assistance Training parameters: One hour per session, ten sessions per therapy cycle which lasted two weeks before subjects switched groups.	Kinematic: Metric indexes (movement smoothness, movement accuracy, path length ratio, movement direction variability) Clinical: MSS^c^, MAS^d^, ROM Shoulder and Elbow	MAS: in the first cycle, ER showed more improvement than EA (3.5 ± 2.8 vs 1.8 ± 3.6), but level of significance not provided. In the second cycle, ER still showed more improvement than EA (0.9 ± 3.5 vs 0.3 ± 2.7), but level of significance not provided. MSS: in the first cycle, ER showed more improvement than EA (2.9 ± 7.1 vs 1.8 ± 5.2), but level of significance not provided. In the second cycle, ER still showed more improvement than EA (1.0 ± 4.8 vs 0.6 ± 6.4), but level of significance not provided. ROMs: no significant changes (numerical values not provided) Metric indexes: no numerical values reported, so unable to calculate differences between groups. The authors reported final metric indexes differences were not significant in the group started with EA (F = 1.61, *p* = 0.194) but in the group started with ER, there was a significant improvement indexes (F = 9,46, *p* = 0.006). They did not mention the comparison of metric indexes between groups.	MAS: first cycle 0.53 (medium effect). second cycle 0.19 (very small effect). MSS: first cycle 0.18 (very small effect). second cycle 0.09 (very small effect).	3/10, poor quality
Patton et al. [24]	Quasi-experimental design. Trial not registered	31 in total, Stroke Group EA = 9 ER = 9 C = 9 Healthy Group EA = 2 ER = 2	Ages = 30–72 years (EA: mean = 54.3 ± 8.8;ER: mean = 48.0 ± 8.4;Control: mean = 51.2 ± 6.1), besides 4 healthy subjects, all participants suffered from a chronic stroke (16–173 months, EA: mean = 69.1 ± 50.2; ER: mean = 109.3 ± 45.8; Control: mean = 70.8 ± 60.4), FM: EA group mean = 40.2 ± 13.7 ER group mean = 25.5 ± 10.9 Control group mean = 37.3 ± 16.2	Free-extremity robot with two degrees of freedom. The participant’s arm was supported by a low-friction, low-impedance mechanism	Experimental tasks: reaching EA group: both stroke and healthy EA groups received force field that magnified errors (EA) ER group: both stroke and healthy ER groups received force field that reduced error. In the stroke control group, the 9 participants with stroke did not receive interfering forces. Training parameters: One single session of 834 movements.	Kinematic: initial direction error (degrees). Adaptation capacity	The stroke EA group showed improvement at initial direction error (8.9 ± 10.9) while the stroke ER group showed deterioration (− 6.8 ± 9.6). The different between EA and ER groups was significant [F(1,13) = 4.29, *p* < 0.001]. Stroke subjects showed less adaptation capacity than healthy subjects (26% less)	Initial direction error: 1.53 (very large effect)	1/10, poor quality
Tropea et al. [54]	Crossover randomized controlled trial. Trial not registered	18 in total EA = 9 ER = 9 (before cross-over)	Ages: 21–71 (EA: mean = 49.7± 18.7; ER: mean = 44.9 ± 15.9), 9 males and 9 females, all participants suffered from a chronic stroke (mean/SD unknown), CM: First EA group: mean = 4.9 ± 0.9 First ER group: mean = 4.2 ± 1.0	InMotion2 robotic system	Experimental tasks: reaching targets in a plane. EA group: received divergent force field ER group: received active assist during practice Training parameters: Two weeks of training per cycle, and two cycles in total. After each cycle, subjects switch groups.	Kinematic: the trajectory of the end-effector Clinical: MAS, MSS	MAS: in the first cycle, ER group showed more improvement than EA (2.9 ± 3.2 vs 1.2 ± 3.2), but not significantly. In the second cycle, ER group still showed more improvement than EA (1.4 ± 1.2 vs 0.7 ± 2.3), but not significantly. MSS: in the first cycle, ER group showed more improvement than EA (2.2 ± 2.0 vs 0.8 ± 3.5), but not significantly. In the second cycle, ER group still showed more improvement than EA (1.4 ± 1.3 vs 1.1 ± 1.1), but not significantly. Trajectory of end-effector: no numerical values reported, but authors stated that EA group had significantly straighter (*p* = 0.028) as well as smoother (*p* = 0.031) trajectory than ER group	MAS: first cycle 0.53 (medium effect) Second cycle 0.40 (small effect) MSS: first cycle 0.51 (medium effect) Second cycle 0.25 (small effect)	6/10, high quality

^a^AMFM: Arm Motor Fugl-Meyer

^b^CM: Chedoke-McMaster scale score

^c^MSS: Motor Status Score

^d^MAS: Modified Ashworth Scale

### Participants

Besides two studies that included healthy subjects in their control groups [24, 60], all other studies only included stroke survivors [52–59, 61–63]. Eleven out of thirteen selected studies [24, 52–57, 59–62] recruited participants with a chronic stroke (i.e. more than six months post-stroke’ [64]) with a mean value of 74.5 ± 46.8 months, one study [63] recruited participants with an acute stroke (i.e. less than a month post-stroke [65]), and one study did not specify in which stage of stroke the participants were situated [58]. The number of participants varied from study to study, ranging from 7 [63] to 34 [61] with a mean value of 19.6. The age of the participants varied greatly among studies, and almost every study included young as well as seniors who were 65 and above. The mean age of all 13 studies is 55.04 ± 11.3 years. In terms of baseline clinical assessments, nine studies used Fugl-Meyer (FM) assessment scores [24, 52, 53, 56, 57, 60–63], three studies used Chedoke-McMaster Stroke Assessment (CM) scores [54, 55, 58], and one did not include any baseline clinical information [59]. Among the studies that used FM scores, five studies included stroke survivors with the average FM scores ranging between 30 and 40 (the lowest being 15 and the highest being 50) [24, 52, 53, 60]. One study [63] recruited participants with stroke with a mean FM score around 53–54, which indicated a higher functional level. Four other studies used the Arm Motor Fugl-Meyer (AMFM) scores which correspond to the upper extremity section of the FM and reported mean AMFM scores between 30 and 40 [62], 40–50 [56], 50–60 [57], and 60–66 [61]. Among the studies that used CM (scores ranging from 1 (lowest) to 7 (highest)), the mean CM stages were respectively 3.3 [55], 4.4 [58], and 4.56 [54].

### Experiment protocols

Among the five crossover studies, two [52, 60] involved a protocol in which participants crossed between experimental intervention (all of them are related to EA paradigm) and control intervention (no distorted error feedback); two studies [54, 58] had participants crossing between EA interventions and ER interventions; one study had participants crossing between EA force alone and EA combined with positive limb inertia [59]. One study [56] divided the participants into two groups, the first one receiving ER throughout the experiment and the second one receiving the control intervention (no assistance) for the first half of the experiment and ER for the second half of the experiment. In the study of Patton and colleagues (2006) there were three groups: stroke experimental, stroke control and healthy experimental. Half of the stroke experimental group experienced EA and the other half experienced ER, but it was unclear which intervention the healthy experimental group received [24]. Rozario and colleagues (2009) also recruited healthy subjects in the study as control group, but likewise, it was unclear which interventions did the healthy subjects receive [60]. The duration of the experiment varied greatly among the studies. Eight studies had protocols that involved multiple sessions over three to eight weeks [52, 54, 56–58, 60, 62, 63]. Four studies only had one single session [24, 53, 55, 61] and one study had three session in total [59].

### Outcomes measures

All studies included clinical outcome assessments except two (Patton et al. 2006; Huang and Patton 2013 [59, 61]). The AMFM and CM impairment inventory were the most frequently used clinical assessment scale, as they were used in nine [24, 52, 53, 55–57, 60, 62, 63] out of eleven studies that included clinical outcome measures. The Box and Blocks Test was used in three studies [52, 56, 60], the Wolf Motor Function Test (WMFT - functional ability scale (FAS) and time measures) in two studies [52, 60], range of motion (ROM) in two studies [52, 58], Motor Status Score (MSS) in two studies [54, 58], Modified Ashworth Scale (MAS) in two studies [24, 58] and Action Research Arm Test (ARAT) in two studies [56, 57]. For data analysis and synthesis purposes, clinical scales are prioritized in the following way: (1) for motor impairments, AMFM>CM > MSS > MAS > ROM; (2) for motor disabilities, WMFT>MAL > Motor Assessment Scale>ARAT>Box and Blocks. Eight studies [24, 53–55, 58, 59, 61, 63] further included kinematic outcomes. While the kinematic outcomes used were different from study to study, most of them were related to spatial, timing or velocity deviation errors [24, 53, 59, 61, 63]. One study used movement accuracy and smoothness as its main kinematic outcome [58], and one study included trajectory of movement [54]. Other kinematic outcomes such as distance of reach [55] and speed of movement [55] were also used. It is to be noted that Takahashi and colleagues (2008) included electromyography (EMG) and functional magnetic resonance imaging (fMRI) as outcome assessment tools, but the results of the imaging techniques were not the focus point of this review and will not be discussed.

### Methodological quality of trials

The information on the agreement between the two reviewers using Cohen’s kappa can be found in Table 4. The mean±1 standard error of Cohen’s kappa of all items of PEDro scale was 0.423± 0.202, which could only be considered “moderate” [66] although the mean observed agreement percentage (P_o_) was high (78.32%). This could be due the fact that the mean expected agreement percentage (P_e_) was 63.15% which is also considered to be medium-high. Table 5 summarizes the final score of PEDro scale of the selected studies after a comparison of results and discussion between the two reviewers. Five studies [52, 54, 57, 61, 62] were considered to be of ‘high quality’ which represents a score of 6/10 or above [47]. Four studies [53, 55, 56, 60] were considered to be of ‘fair quality’ which indicates a score between 4/10 and 5/10 [47]. At last, four studies [24, 58, 59, 63] were considered of ‘poor quality’ due to having a score less than 4/10 [47]. The parameters that received the lowest scores were ‘blinded therapists’ (one out of fourteen studies), ‘concealed allocation’ (two out of fourteen studies), and ‘intention to treat analysis’ (three out of fourteen studies). Total scores on the quality of trials were also included in Tables 1, 2 and 3.

**Table 4:** Assessment of agreement among the reviewers on the ratings of PEDro scale using Cohen’s kappa

	Eligibility criteria specified	Randomized allocation	Concealed allocation	Baseline similarity	Blinded subjects	Blinded therapists	Blinded assessors	Adequate follow-up	Intention to treat analysis	Comparison between groups	Point estimates and variability
Kappa (mean/SE)	0.629 ± 0.331	0.755 ± 0.228	− 0.083 ± 0.059^a^	0.567 ± 0.273	0.270 ± 0.234	0.316 ± 0.253	0.698 ± 0.187	0.025 ± 0.212	0.316 ± 0.175	1.000 ± 0.000	0.161 ± 0.275
95% Confidence Interval (CI)	−0.021 to 1.000	0.307 to 1.000	−0.198 to 0.032	0.032 to 1.000	−0.190 to 0.729	− 0.179 to 0.811	0.330 to 1.000	− 0.390 to 0.440	−0.027 to 0.659	1.000 to 1.000	−0.378 to 0.700
Observed agreement percentage (P_0_)	92.31%	92.31%	84.62%	84.62%	61.54%	76.92%	84.62%	53.82%	61.54%	100.00%	69.23%
Expected agreement percentage (P_e_)	79.29%	68.64%	85.80%	64.50%	47.34%	66.27%	49.11%	52.66%	43.79%	73.96%	63.31%

^a^The kappa score for this item was negative despite having a high number of agreement. This occurred because the expected agreement percentage was greater than the observed agreement percentage

**Table 5:** Methodological quality assessment of the studies using PEDro scale

	Eligibility criteria specified	Randomized allocation	Concealed allocation	Baseline similarity	Blinded subjects	Blinded therapists	Blinded assessors	Adequate follow-up	Intention to treat analysis	Comparison between groups	Point estimates and variability	Total
Abdollahi et al. [52]	Yes	1^a^	0	0	1	1	1	0	1	1	1	7/10^b^
Bouchard et al. [61]	Yes	1	0	1	1	0	1	1	1	1	1	8/10
Cesqui et al. [58]	Yes	0	0	0	1	0	0	0	0	1	1	3/10
Givon-Mayo et al. [63]	Yes	1	0	0	1	0	0	0	0	1	0	3/10
Huang and Patton [59]	No	1	0	0	0	0	0	0	0	1	1	3/10
Kahn et al. [55]	Yes	1	0	0	0	0	1	0	0	1	1	4/10
Majeed et al. [62]	Yes	1	0	0	1	0	1	1	0	1	1	6/10
Patton et al. [53]	Yes	1	0	0	1	0	1	1	0	0	1	5/10
Patton et al. [24]	Yes	0	0	0	0	0	0	0	0	1	0	1/10
Rozario et al. [60]	Yes	1	0	0	1	0	1	1	0	0	0	4/10
Takahashi et al. [56]	Yes	1	0	1	1	0	0	1	0	1	0	5/10
Timmer-mans et al. [57]	Yes	1	1	1	0	0	1	1	1	1	1	8/10
Tropea et al. [54]	Yes	1	0	1	0	0	1	1	0	1	1	6/10

^a^Items that were not reported were scored as 0, and reported items were scored as 1. Evaluation was conducted by two reviewers. ^b^Interpretation of scores: high quality- 6 points or more, fair quality- 4-5 points, poor quality- less than 4 points

### Assessment of risk of bias

The risk of bias of the selected studies was assessed using Cochrane Collaboration’s risk of bias tool (Table 6). It is to be noted that two studies [24, 63] had high risk of bias in four of the six domains and four studies [55, 56, 58, 59] were considered of having high risk of bias in three of the six domains. The domain that received the highest risk of bias is ‘allocation concealment’ (twelve out of fourteen studies). In the domain of ‘other bias’, two most common biases were ‘small sample size’ which was present in seven of the thirteen studies [53–56, 58, 60, 63] as well as ‘short training protocol’ which was found in five of the thirteen studies [24, 53, 55, 59, 61].

**Table 6:** Assessment of risk of bias of the studies using Cochrane Collaboration’s risk of bias tool

	Random sequence generation (selection bias)	Allocation concealment (selection bias)	Blinding of participants and personnel (performance bias)	Blinding of outcome assessment (detection bias)	Incomplete outcome data (attrition bias)	Selective reporting (reporting bias)	Other bias
Abdollahi et al. [52]	Low^a^	High^b^	Low	Low	Low	Low	Low
Bouchard et al. [61]	Low	High	Low	Low	Low	Low	Short training period
Cesqui et al. [58]	High	High	Unclear^c^	High	High	High	Small sample size, baseline differences between groups
Givon-Mayo et al. [63]	Low	High	High	High	High	Unclear	Very small sample size, baseline differences between groups
Huang and Patton [59]	Low	High	High	High	Low	Low	Short training period
Kahn et al. [55]	Low	High	High	Low	Unclear	High	Small sample size, short training period
Majeed et al. [62]	Low	High	Low	Low	Low	Low	Low
Patton et al. [53]	Low	High	High	Low	Low	Low	Short training period, small sample size
Patton et al. [24]	High	High	High	High	Low	Low	Short training period
Rozario et al. [60]	Low	High	High	Low	Low	Low	Small sample size
Takahashi et al. [56]	Low	High	Low	High	High	Low	Small sample size
Timmermans et al. [57]	Low	Low	High	Low	Low	Low	Low
Tropea et al. [54]	Low	High	High	Low	Low	High	Small sample size

^a^Low: low risk of bias; ^b^High: high risk of bias; ^c^Unclear: unclear risk of bias

### Data analysis and synthesis

#### EA compared to training without error modification

As shown in Table 1, two high quality [52, 62], two fair quality [53, 60] and two poor quality [59, 63] studies investigated the effectiveness of EA compared to standard repetitive practice. In the first high quality RCT of Abdollahi and colleagues (2014) [52], the EA group showed significantly higher improvement with a medium effect size over the control group in AMFM score during the first phase of training. In the second phase, the difference was of low effect size and not significant [52]. When examining the results of WMFT FAS, the EA group showed higher improvement in the first phase, but the opposite was seen in the second phase [52], and this might be due to the EA training having a stronger cross-over effect. The effect size of both phases were medium, but the levels of significance were unknown. The results of WMFT timing measures were in favor of the EA group in both phases, but the effect sizes were lo*w*/*v*ery low and the levels of significance were unknown. In the Box and Block Test, no significant difference was found [52]. In the second high quality study of Majeed and colleagues (2015) [62], the AMFM scores were not found to be different between the EA and control group. It is to be noted that in this study, the training period was considerably shorter than the one in Abdollahi et al. (2014). However, the EA group showed significantly better retention in AMFM at one week follow-up with a medium effect size [62].

In the two fair quality studies, Patton and colleagues (2006) and Rozario and colleagues (2009) [53, 60], the EA group showed higher improvement than the control group in movement and ROM errors. The effect sizes were medium, but the levels of significance were unknown (possibly insignificant because the sample sizes of the two studies were small: 15 and 10).

In the pilot study of Givon-Mayo and colleagues (2014) [63], the EA group showed higher improvement of medium effect size over the control group in Motor Assessment Scale scores, but the level of significance was unknown (possibly insignificant because the sample size was really small: 7). It was demonstrated that the EA group also improved greatly over the control group in velocity deviation error (a measure of velocity error expressed as deviation from the optimal smooth acceleration), and the result had a very large effect size and was significant [63]. In the study of Huang and Patton (2013), the EA group was the only group to have a significant improvement in radial deviation (a measure of movement error expressed as the distance between handle and template track in a circular movement task) compared to the control and the EA combined with inertia groups, though the effect size was small [59].

In summary, the following conclusions were drawn:

1. There is *moderate evidence (Level 1b)* from one high quality study [52] that the EA training paradigm is *more effective* than standard repetitive practice without error modification at improving upper extremity motor impairments (as measured by AMFM) among people with chronic stroke.
2. There is *moderate evidence (Level 1b)* from one high quality study [62] that the EA training paradigm shows *more retention of improvement* than standard repetitive practice without error modification for upper extremity motor impairments (as measured by AMFM) among people with chronic stroke.
3. There is *moderate evidence (Level 1b)* from one high quality study [52] and one pilot study [63] that the EA training paradigm is *more effective* than standard repetitive practice without error modification at improving upper extremity functional disability (as measured by WMFT and Motor Assessment Scale) among people with chronic stroke.
4. There is *limited evidence (Level 2a)* from two fair quality studies [53, 60], one pilot study [63], and one poor quality study [59] that EA training paradigm *is more effective* than standard repetitive practice without error modification at improving reaching trajectory deviation and control (measured by kinematic outcomes such as movement errors, velocity errors, etc) among people with chronic stroke.

#### ER compared to training without error modification

One high quality RCT [57] and two fair quality RCTs [55, 56] were included when comparing ER to training without error modification (Table 2). In the high quality study of Timmermans and colleagues (2014) [57], the control group consistently showed more improvement than the ER group at every outcome measure (AMFM, ARAT, and Motor Activity Log), but the differences in scores between the two groups were not significant and the effect sizes were either small or very small.

In the fair quality study of Kahn and colleagues (2006) [55], the ER group showed more improvement than the control group in supported fraction of range (the reaching range of the affected arm, while supported by the robotic device, normalized to the same measure of the unaffected side) and supported fraction of speed (the reaching speed of the affected arm normalized to the same measure of the unaffected side), but the opposite result was seen in unsupported fraction of speed (the reaching speed of the affected arm without the support of the robotic device) and CM assessment. All results in the study had small or very small effect sizes, and none was significant [55]. However, in another fair quality study of Takahashi and colleagues (2008), the full ER group had higher improvement of very large effect size over the half ER/half control group at ARAT and AMFM scores, and the differences were significant [56]. In that same study, no change was found in the Box and Block Test.

The following conclusions were drawn:

1. There is *moderate evidence (Level 1b)* from one high quality study [57] that the ER training paradigm is *not more effective* than standard repetitive practice without error modification at improving upper extremity motor impairments (as measured by AMFM) or at improving upper extremity functional disability (as measures by ARAT and MAL) among people with chronic stroke.
2. There is *limited evidence (Level 2a)* from one fair quality study [55] that ER training paradigm *is not more effective* than standard repetitive practice without error modification at improving reaching trajectory control (measured by kinematic outcomes such as supported range and supported speed) among people with chronic stroke.

#### EA compared the ER

Two high quality studies [54, 61] as well as two poor quality studies [24, 58] were included in the analysis (Table 3). In the high quality study of Bouchard and colleagues (2016) [61], the ER group had an improvement in absolute timing errors while the EA group had a deterioration, but the difference between the two groups was not significant and the effect size was small. In the high quality study of Tropea and colleagues (2013) [54], the ER group had a non-significant difference of improvement in Modified Ashworth Scale (MAS) and Motor Status Score (MSS) compared to the EA group, and the effect sizes were small to medium. However, the EA group had a significantly smoother and straighter trajectory than the ER group [54].

In the study of Cesqui and colleagues (2008) [58], similar results were found in terms of difference between EA and ER groups in MAS and MSS as in the study of Tropea et al. (2013). In the quasi-experimental study of Patton and colleagues (2006), the EA group showed a very large effect size at improvement in initial direction error over the ER group, and the result was significant [24].

The following conclusions were drawn:

1. There is *moderate evidence (Level 1b)* from one high quality study [54] that the EA training paradigm is *not more effective* than the ER training paradigm at improving upper extremity spasticity (as measured by MAS) and motor impairment (as measured by MSS) among people with chronic stroke. It is to be noted however, that in this study the baseline stroke severity between the two groups was different.
2. There is *moderate evidence (Level 1b)* from one high quality study [61] that the EA training paradigm is *not more effective* than ER training paradigm at improving movement timing (measured by absolute timing error) during a wrist flexion movement among people with chronic stroke.
3. There is *moderate evidence (Level 1b)* from one high quality study [54] and one quasi-experimental study [24] that the EA training paradigm is *more effective* than ER training paradigm at improving reaching trajectory control (as measured by kinematic outcomes such as trajectory smoothness, straightness and initial direction errors) among people with chronic stroke.

Overall, results suggested that EA induces larger improvement in clinical and kinematic outcomes compared to standard repetitive practice without error modification. Furthermore, results also unveiled the new findings that (i) there is a lack of evidence supporting the superiority of ER over standard repetitive practice in terms of improvement in clinical and kinematic outcomes; and (ii) EA is only superior to ER at improving kinematic outcomes. These findings were supported, globally, with a moderate level of evidence.

## Discussion

This study completed, for the first time, a systematic review of interventions studies that compared the effectiveness of the EA training paradigm to standard repetitive practice without error modification, the ER paradigm to standard repetitive practice, and EA to ER at enhancing upper extremity motor recovery and performance in individuals with stroke. Thirteen studies were included in the review. The reason why EA was found to more effective than standard repetitive practice while ER was not could be due to the fact haptic guidance and assistive therapy are more effective in the initial stage of motor learning while error-based learning is more used in the later stage of learning. Indeed, it has been shown that in the initial stage of motor learning, motivation and positive reinforcement are believed to play a much more important role than being able to identify errors [28]. Since most participants in the reviewed studies are people with chronic stage of stroke, it is believed that they have already gone through the initial stage of motor relearning.

While some differences in clinical outcomes between training paradigms were statistically significant, it is also important to assess their clinical relevance and effect size in order to address the objectives of this review. Amongst clinical tests that assess motor recovery, the AMFM shows a minimal detectable change (MDC) of 5.2 [67] and a minimally clinically important difference (MCID) of ranging from 4.25 to 7.25 [68]. None of the reviewed studies on EA presented intervention gains that met the MDC or MCID for this test. In fact, only Takahashi and colleagues (2014) [56] who compared ER to standard practice had results that met the MDC and MCID for the AMFM, in both intervention groups. For the WMFT FAS and the WMFT time measure which reflect motor abilities in functional and timed tasks, none of the studies reviewed met the MCID (WMFT FAS ranging from 0.2 to 0.4 point; WMFT time measure ranging from 1.5 to 2.0 s [69]). The MCID for the ARAT (5.7 [70]) was attained only in Timmermans and colleagues’ study (2014) [57], both by the ER and standard practice groups. It is to be noted that no established MCID was found in Motor Assessment Scale, Motor Activity Log and Motor Status Score. Spasticity, as measured by the MAS, showed intervention induced changes that reached the MCID (1 point [71]) for ER and EA in two studies that compared the latter two approaches [54, 58]. The Box and Blocks test and ROM did not see any significant change in any of the intervention groups in the thirteen studies reviewed, presumably because arm trajectory control was specifically targeted in the interventions, as opposed to manual dexterity and joint mobility. In addition, the effect sizes of the differences in clinical outcomes in all thirteen studies were for most moderate or small. Collectively, these observations suggest that while EA was found to have superior effects over standard repetitive practice to improve upper extremity motor impairments and functional disability, it yet has to demonstrate that it can yield clinically meaningful changes in clinical outcomes of motor impairment and function. Such observations also raise important questions, being whether the intervention was delivered optimally (e.g. in terms of training intensity, duration, feedback sensory modality, stroke chronicity and baseline level of motor recovery, etc.) and whether the selected outcomes were actually best suited to capture the improvements brought up by the intervention.

To that effect, the EA training paradigm was further found to be more effective at improving kinematic outcomes that measure reaching trajectory control compared to both ER and standard repetitive practice. Indeed, two studies showed very large effect sizes on the difference between EA and standard repetitive practice, and between EA and ER [24, 63]. Furthermore, when comparing EA to ER, the only statistically significant difference that emerged was in the kinematic outcomes which were in favor of the EA group. In fact, although EA showed larger improvement than standard practice and although ER did not show significant difference compared to standard practice in terms of clinical outcomes, EA surprisingly did not appear to be better than ER at improving clinical outcomes. It has been shown that kinematic variables are highly responsive to changes in motor performance following training intervention [72] and that they can capture the quality of the movement which is another important aspect of motor abilities [73]. In the context of this study, this could suggest that EA is actually better than ER at improving the quality of movement which is mostly measured by the kinematic outcomes, but such improvement could not be detected by most of the examined clinical outcomes. From a broader perspective, these observations emphasize the need to deeply understand the mechanisms of action of error modification interventions and select outcome measures accordingly.

Besides factors related to the intervention itself (intensity, duration, etc), personal-related factors such as the site of lesion, stroke severity and chronicity also are factors that may have influenced the results of studies reviewed in this manuscript and ensuing conclusions. Unfortunately, most studies did not provide information on brain lesion location. Among the three studies that did provide this information [24, 52, 53], participants suffered stroke in a variety areas (e.g. cortical, sub-cortical, thalamus, basal ganglia, brain stem, etc.) and the distribution of the different sites of lesion amongst groups was not reported, making it impossible to analyse the effects of lesion location. As for stroke severity, among the studies that compared EA to repetitive practice, baseline AMFM scores did not seem to influence the results because participants who had AMFM scores ranging from 15 to 55 [52, 53, 60, 62, 63] all demonstrated larger improvement with the EA training. However, it was difficult to draw definite conclusions on ER vs. standard repetitive practice and EA vs. ER, as the number of studies in these two categories was small and studies used different outcome measures to assess stroke severity. Lastly, most of the studies only recruited chronic stroke survivors, making it difficult to appraise the effects of stroke chronicity while limiting the generalization of findings mainly to chronic stroke survivors.

Results of this review also highlighted contradictions across studies which could be due to an influence of participants’ personal factors on intervention outcomes. For instance, Takahashi and colleagues (2008) [56] suggested that full ER practice was better than half ER/half standard repetitive practice at improving AMFM and ARAT scores, a finding that was in contradiction with that of other studies [55, 57]. The full ER group, however, had an average onset of stroke of 1.2 years compared to 4.8 years for the other intervention group, and this suggests that time of stroke onset might be a factor that influences the motor recovery [56]. Moreover, the full ER group also had nine points less in baseline average AMFM scores compared to the other group [56], possibly leaving more room for improvement in the former group. We therefore suggest that at this point in time, a deeper investigation of patient-related factors on the intervention outcomes is warranted.

This systematic review has some limitations. The risk of bias among the selected studies is high as most of the selected studies have either short training period or small sample size. Another limitation lies in the fact that many studies did not provide numerical values for the standard deviations of their results, or the standard deviations had to be estimated from tables or figures, which may have affected the calculation of some effect sizes. Only one out of 13 studies [57] reported the effects of intervention on the arm use which is an important predictor of upper extremity motor recovery. It should also be noted that 6 out of 10 studies involving EA trainings may come from the same research group [24, 52, 53, 59, 60, 62]. Moreover, the main methodological quality assessment was done using the PEDro scale. Like many checklist-style appraisal tools, PEDro has a disadvantage of giving the same weighing (1 point) to every category of source of bias. However, depending on the types of study, not all sources of bias affect the internal validity equally. Finally, before starting this systematic review, the authors have planned to conduct experimental studies on the use of EA and ER on motor learning in the future, therefore this could act as a source of bias, although unwillingly.

## Conclusion

In response to the research questions posed in this paper, the following conclusions were drawn with regards to the population of *chronic stroke*: (1) Interventions involving an EA paradigm were more effective compared to interventions without error modification at improving upper extremity impairments, disabilities and reaching trajectory control; (2) Interventions involving ER paradigm were not more effective compared to interventions without error modification at improving upper extremity impairments and disabilities and; (3) Interventions involving an EA paradigm were more effective compared to interventions involving an ER paradigm to improve reaching trajectory control. While these conclusions hold true at a statistical level, however, this review further demonstrates that EA and ER, like standard repetitive practice, induced changes in clinical outcomes of motor recovery and function that did not reach the minimal clinically important difference. Nevertheless, this review showed that EA paradigm has promising effects for post-stroke upper extremity rehabilitation.

In the future, clinical trials of strong methodological quality which include sensitive outcomes that capture changes in movement quality and patient functioning in activities of daily living are needed to further demonstrate the effects of error-modification therapies with a stronger level of evidence and to possibly achieve clinically meaningful changes. The influence of intervention-related factors such as training intensity and duration, as well as personal factors such as the site of lesion, severity of stroke and stroke chronicity on the error-modification intervention paradigms should further be explored. Finally, the emergence of virtual reality makes other modalities, namely visual and auditory feedback, potential alternatives to haptic feedback. These modalities could be cheaper and easier to implement than robotics, and it appears that more and more studies have begun to examine the effect of these feedback on motor learning. Therefore, the use of different modalities of feedback, such as visual, auditory and/or a combination of multiple sensory modalities, could also be investigated.

## Figures and Tables

**Fig. 2:**
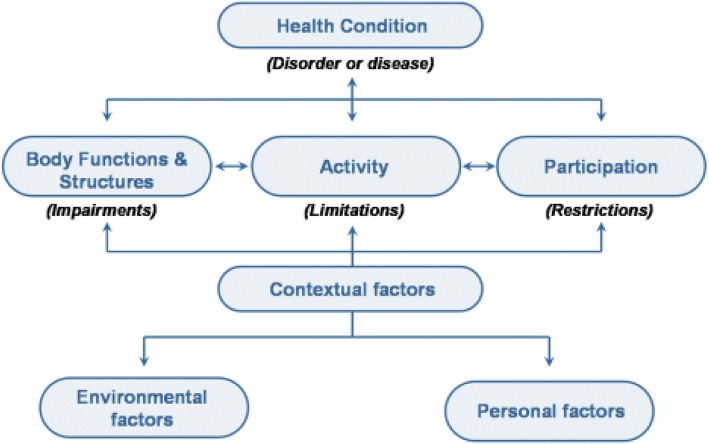
ICF model. Figure modified from WHO [74]

## Appendix 2

**Table 7:** Articles excluded following full-text review

Abdollahi et al. (2011) [75]	This study is a pilot study of Abdollahi et al. (2014) [52].
Agostini et al. (2011) [76]	This study lack of control group.
Arab Baniasad et al. (2014) [77]	It is a conference abstract, possibly lack of control group as well.
Badia et al. (2008) [78]	It is the same study as the one of Cameirao et al. (2008)
Basteris et al. (2014) [79]	It is a review
Beling et al. (2015) [80]	It is a conference abstract, and it does not have the intervention of interest
Broeren et al. (2007) [81]	It does not have the intervention of interest.
Cameirao et al. (2012) [82]	It does not have the intervention of interest.
Cameirao et al. (2008) [83]	It is a review.
Casadio et al. (2009) [84]	It does not have the intervention of interest and lacks of a control group.
Chemuturi et al. (2013) [85]	This study only recruited healthy subjects and it does not have an the intervention of interest.
Chemuturi et al. (2013) [86]	This study by the same authors as above also only recruited healthy subjects and it does not have an the intervention of interest.
Coote et al. (2008) [87]	This study lacks a control group of interest.
Crocher et al. (2012) [88]	This study does not have interventions of interest or results of interest.
De Santis et al. (2015) [89]	This study does not have a control group and it lacks outcomes of interest.
Fasoli et al. (2004) [90]	This study does not have a proper control group.
Fischer et al. (2016) [91]	This study does not have the intervention of interest.
Fluet et al. (2011) [92]	It is a conference abstract.
Fluet et al. (2012) [93]	It is a book.
Fluet et al. (2014) [94]	This study does not have the intervention of interest.
Hachisuka et al. (2014) [95]	It is a conference abstract.
Housman et al. (2009) [96]	It is the same study as the one of Rozario et al. (2009) [60].
Huang and Patton(2011) [97]	It has the same results as the one of Huang and Patton(2013) [59].
Israely and Carmeli (2016) [40]	It is a review.
Krebs et al. (2008) [98]	This study does not have the intervention of interest.
Lam et al. (2008) [99]	This study only has healthy subjects, and it lacks of the intervention of interest.
Lambercy et al. (2011) [100]	This study lacks of a control group.
Lemmens et al. (2012) [101]	It is a conference abstract.
Liao et al. (2012) [102]	This study does not have the intervention of interest.
Lin et al. (2015) [103]	This study does not have the intervention of interest.
Milot et al. (2016) [104]	This paper is about design and implementation, and not intervention-oriented.
Oblak et al. (2010) [105]	This paper is not intervention-oriented.
Orihuela-Espina et al. (2016) [106]	This study does not have the intervention of interest.
Patton and Mussa-Ivaldi (2004) [107]	This study only recruited healthy subjects.
Perry et al. (2011) [108]	This study is not intervention-oriented.
Phyo et al. (2016) [109]	This study lacks of the intervention and results of interest.
Squeri et al. (2014) [110]	This study lacks of a control group.
Stein et al. (2004) [111]	This study contains subjects under 21 years old.
Timmermans et al. (2012) [112]	It is a conference abstract.
Timmermans et al. (2012) [113]	It is another conference abstract by the same author as above.
Turolla et al. (2013) [114]	This study does not have a control group.
Waldner et al. (2009) [115]	This study is not intervention-oriented.
Ziheri et al. (2010) [116]	This study does not have the intervention or control of interest.
Zondervan et al. (2013) [117]	This study does not have the intervention of interest.

## Appendix 3

**Table 8:** Ratings of level of evidence from Evidence Based Medicine

Level of evidence	
*Evidence level*	Study type
*1a* (Strong)	Well-designed meta- analysis, or 2 or more “high” quality RCTs (PEDro Scale scores ≥ 6) that show similar findings
1b (Moderate)	One RCT of “high” quality (PEDro Scale score ≥ 6)
2a (Limited)	At least one “fair” quality RCT (PEDro Scale score = 4–5)
2b (Limited)	At least one well-designed non-experimental study: Non-randomised controlled trial; quasi-experimental studies; cohort studies with multiple baselines; single subjects series with mutiple baselines
3 (Consensus)	Agreement by an expert panel, a group of professionals in the field or a number of pre-post design studies with similar results
4 (Conflicting)	Conflicting evidence of two or more equally designed studies
5 (No Evidence)	No well-designed studies: “Poor” quality RCTs with PEDro scores ≤ 3; only case studies/case descriptions, or cohort studies/single series with no multiple baselines)

Table taken from *Sackett (2000)* [51]

## Abbreviations

ADLs: Activities of daily living
AMFM: Arm motor Fugl-Meyer
ARAT: Action Research Arm Test
CM: Chedoke-McMaster scale score
CVA: Cerebrovascular accident
EA: Error augmentation
ER: Error reduction
FM: Fugl-Meyer Assessment of Motor Recovery after Stroke
ICF: International Classification of Functioning, Disability and Health
MAL: Motor Activity Log
MAS: Modified Ashworth Scale
MCID: Minimally clinically important difference
MDC: Minimal detectable change
MSS: Motor Status Score
ROM: Range of motion
WMFT FAS: Wolf Motion Function Test Functional Ability Scale
